# Impact of perioperative enteral nutrition on postoperative acute muscle wasting in gastric cancer: a prospective exploratory study with a historical control group

**DOI:** 10.3389/fonc.2025.1607756

**Published:** 2025-07-11

**Authors:** Xing Zeng, Xuan Ni, Zhili Shen, Dong Yang, Yajing Gu, Ai Li, Hui Liu, Changdi Li

**Affiliations:** ^1^ Department of Gastrointestinal Oncology Surgery, The Affiliated Jiangning Hospital of Nanjing Medical University, Nanjing, China; ^2^ Chinese Hospital Reform and Development Institute, Nanjing University, Nanjing, China; ^3^ Department of Orthopedics; The Affiliated Jiangning Hospital of Nanjing Medical University, Nanjing, China; ^4^ Department of Urology. The Affiliated Jiangning Hospital of Nanjing Medical University, Nanjing, China; ^5^ Nursing Department, The Affiliated Jiangning Hospital of Nanjing Medical University, Nanjing, China

**Keywords:** gastric cancer, perioperative period, enteral nutrition, postoperative acute muscle wasting, management

## Abstract

**Objective:**

Assessing the impact of perioperative enteral nutrition (EN) on postoperative acute muscle atrophy following radical gastrectomy for gastric cancer(GC) (with changes in skeletal muscle mass as the primary outcome indicator).

**Methods:**

Patients who underwent GC surgery at the Department of Gastrointestinal Oncology Surgery in a top-tier hospital in Nanjing were selected for the study. The control group, consisting of patients treated between January and June 2023, received routine perioperative nutritional management. The experimental group, consisting of patients treated between July and December 2023, followed a preoperative combined with early postoperative EN program. skeletal muscle mass, grip strength, 6-meter walk test speed, and body weight were compared between the two groups 7 d postoperatively.

**Results:**

The intervention significantly reduced the loss of skeletal muscle mass, grip strength, and body weight from baseline (*p*<0.01). However, no significant differences in 6-meter walk test speed were observed between the two groups. After adjusting for confounding factors such as age, gender, nutritional risk screening 2002(NRS 2002) score, education level, diabetes comorbidity, tumor staging, surgical approach, intraoperative blood loss, and operation time, multivariate linear regression analysis showed that the EN program independently influenced the loss rates of skeletal muscle mass, grip strength, and body weight (*p*<0.01).

**Conclusion:**

The perioperative EN program for GC developed in this study enables medical staff to efficiently gather relevant information, providing a more comprehensive and holistic approach to EN for GC patients. The program effectively reduces postoperative acute muscle wasting, grip strength loss, and weight loss. This study provides a reference for clinical perioperative EN management in GC patients.

## Introduction

Gastric cancer persists as a critical global public health challenge, characterized by substantial unmet needs in prevention and therapeutic management. According to the 2024 global cancer statistics released by the International Agency for Research on Cancer (IARC), gastric cancer accounted for 968,000 new cases worldwide in 2022, ranking as the fifth most prevalent malignancy, with 659,900 associated deaths (1). Surgery, serving as the foundational pillar of gastric cancer management, has demonstrated unequivocal efficacy in enhancing long-term patient survival rates. However, the postoperative stress response (2) can cause metabolic and physiological disorders, potentially leading to inflammation, hormonal imbalances, and genomic reactions. These effects contribute to excessive metabolic catabolism and postoperative acute muscle wasting, adversely influencing both short-term postoperative outcomes and long-term prognosis.Currently, there is no standardized definition for postoperative acute muscle wasting. Huang et al. (3) defined the condition as a >10% reduction in total abdominal muscle area (TAMA) measured at postoperative day (POD) 7. In contrast, Naoaki et al. (4) identified a median reduction rate of 4.4% in the total psoas major muscle mass index (TPI) measured at POD 3 as the critical threshold thereby stratifying patients into mild and severe acute muscle wasting. Huang et al. (3). demonstrated that patients exhibiting this early muscle loss experienced significantly elevated fatigue levels and reduced quality of life at both 1-month and 3-month postoperative assessments. Furthermore, these individuals showed higher complication rates, prolonged hospitalization, and increased medical costs particularly when accompanied by a ≥10% reduction in grip strength. Critically, patients with severe postoperative muscle wasting experience pronounced severe side effects from receiving adjuvant chemotherapy than patients without significant muscle depletion (5). Postoperative acute muscle wasting is widely recognized as a significant concern (6), and muscle mass as a nutritional assessment index has drawn considerable interest (7). Patients undergoing gastrectomy for GC must maintain adequate nutrition before surgery to prevent severe weight loss and muscle mass depletion after surgery. Studies have shown that early EN after surgery can reduce the loss of upper and lower limb lean body mass in elderly patients with GC after 8 d (8). However, another study has indicated that preoperative EN exerts no significant effect on muscle mass in patients with esophageal cancer (9). Currently, studies on EN support for GC patients are mostly limited to preoperative or postoperative phases and report less on comprehensive perioperative management. The effectiveness of a comprehensive perioperative EN intervention, combining preoperative and postoperative phases, in reducing postoperative acute muscle wasting in GC remains inconclusive (10). Although the ESPEN guidelines recommend perioperative nutritional support, there are still significant differences in the specific implementation strategies of EN (11–13). The importance of perioperative nutritional management in the postoperative recovery of GC patients has been widely recognized. However, there remains a significant research gap in the intervention strategies for postoperative acute muscle wasting, a common complication (14, 15). This prospective exploratory study, by establishing a historical control cohort, aims to systematically evaluate the impact of standardized perioperative EN support on postoperative acute muscle wasting in GC patients. The findings of this study will provide new evidence-based insights to optimize the perioperative nutritional management pathway for GC patients.

## Materials and methods

### Study design and patients

**Figure 1:**
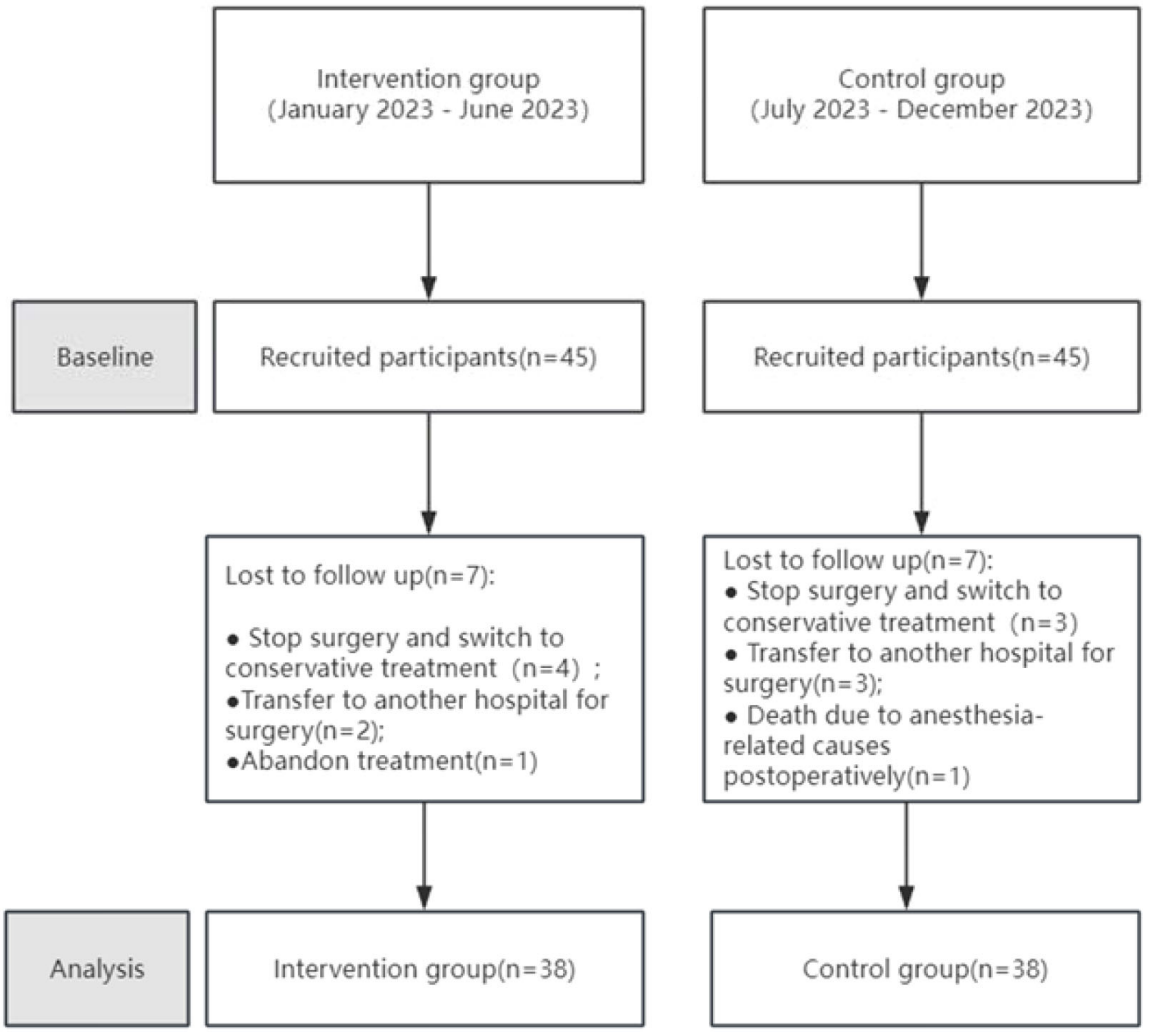
Flowchart of patient enrollment and exclusion.

This study was conducted in accordance with the Declaration of Helsinki and was approved by the Affiliated Jiangning Hospital of Nanjing Medical University (Project number: 2021-03-053-K01), and registered in the Chinese Clinical Trial Registry on August 2, 2023 (Registration number: ChiCTR2500097933). All patients signed informed consent forms and agreed to the use of research data for academic publication. Convenience sampling was used to select GC surgery patients hospitalized at our institution from January to December 2023 as the research subjects. Inclusion criteria were as follows: (1) Patients aged 18–80 y; (2) A clear diagnosis of GC before surgery, based on endoscopy, imaging, and pathological examination, with tumor staging determined according to the 8th edition of the American Joint Committee on Cancer (AJCC) Staging System; (3) Patients who underwent elective radical or palliative gastrectomy in our department; (4) Patients who could cooperate in completing nutritional assessment and supplying basic information. Exclusion criteria were as follows: (1) Patients participating in or who have participated in other clinical studies within the past year; (2) Patients with severe infections, respiratory insufficiency, or other conditions that impair active cooperation; (3) Patients with cognitive impairments, language barriers, or other conditions affecting communication; (4) Patients with hepatic and renal insufficiency; (5) Family members of the research staff involved in the project; (6) Bedridden patients who cannot have their weight measured; (7) Pregnant or lactating women; (8) Individuals unwilling to provide informed consent. Patients who underwent GC surgery and were admitted between January and June 2023 were assigned as the control group, whereas those admitted between July and December 2023 were designated as the experimental group. Initially, 45 patients were included in both the experimental and control groups. Among the 90 enrolled patients, 76 participants were included in the final analysis after excluding those who withdrew or had missing data (**Table 1**). Reasons for withdrawal included death; cessation of surgery and switching to conservative treatment; transfer to another hospital for surgery; and discontinuation of treatment (**Figure 1**).

**Table 1:** Demographic and clinical characteristics of gastric cancer surgery patients in pre- and post-implementation cohorts.

Variable	Control group (n=38)	Intervention group (n=38)	*t/z/χ^2^*	p-value
Age (years), median (IQR)	69.0(58.0,75.0)	70.5(60.8,73.0)	-0.411	0.681^b^
Height (cm, $\bar{x}\pm s$ )	164.2±6.9	162.9±6.9	-0.812	0.419^a^
Weight (kg, $\bar{x}\pm s$ )	60.6±10.4	62.8±10.1	0.927	0.357^a^
skeletal muscle mass (kg, $\bar{x}\pm s$ )	24.8±3.9	25.2±4.2	-0.393	0.695 ^a^
Grip strength (kg, $\bar{x}\pm s$ )	24.6(21.3,28.3)	24.7(21.9,30.8)	-0.857	0.391 ^b^
6-meter walking speed (m/s), median (IQR)	1(0.9,1.1)	1(0.9,1.2)	-0.843	0.399 ^b^
BMI (kg/m^2^, $\bar{x}\pm s$ )	22.4±3.1	23.6±3.2	1.672	0.099^a^
NRS2002 score, median (IQR)	2(1.0,3.0)	2(1.0,3.0)	-0.444	0.657^b^
Gender [n(%)]				
Male	29(76.3)	25(65.8)	1.024	0.312^c^
Female	9(23.7)	13(34.2)		
Education level [n(%)]				
High school and technical secondary school	35(92.1)	35(92.1)	<0.001	1.000^b^
High school and technical secondary school	2(5.3)	2(5.3)		
College and above	1(2.6)	1(2.6)		
Diabetes comorbidity [n(%)]				
Yes	9(23.7)	6(15.8)	0.748	0.387^c^
No	29(76.3)	32(84.2)		
Tumor staging [n(%)]				
IA	3(7.9)	2(5.5)	-1.293	0.196^b^
IB	7(18.4)	4(10.5)		
IIA	7(18.4)	6(15.8)		
IIB	5(13.2)	3(7.9)		
IIIA	8(21.1)	12(31.6)		
IIIB	4(10.5)	7(18.4)		
IIIC	1(2.6)	3(7.7)		
IV	3(7.9)	1(2.6)		
Pathology type [n(%)]				
Unclassified carcinoma	0(0)	1(2.6)		0.564^d^
Signet ring cell carcinoma	1(2.9)	1(2.6)		
Neuroendocrine tumor combined with adenocarcinoma	0(0)	2(5.3)		
Adenocarcinoma	32(91.4)	29(76.3)		
Adenocarcinoma combined with signet ring cell carcinoma	2(5.7)	4(10.5)		
Squamous cell carcinoma	0.(0)	1(2.6)		
Surgical approach [n(%)]				
Laparoscopic surgery	29(76.3)	31(81.6)	3.224	0.073^c^
Laparotomy	9(23.7)	7(18.4)		
Intraoperative blood loss (ml), median (IQR)	200(160.0,240.0)	200.0(160.0,226.3)	-0.100	0.921^b^
Operation time (min), median (IQR)	197.5(160.0,251.3)	175.0(149.8,211.3)	-1.482	0.138^b^

a represents the t-test, b denotes the Mann-Whitney U test, c signifies the Pearson chi-square test, and d indicates the Fisher's exact test.

The control group received routine perioperative nutritional management, while the intervention group underwent a standardized protocol integrating preoperative nutritional optimization with early postoperative EN initiation (detailed in **Table 2**).Additionally, both groups received parenteral nutrition(PN) support at 850kcal/d during postoperative days 1 to 5. This program was implemented by a multidisciplinary team. For quality control, the head nurse of the department organized the training and interpretation of the implementation details of the program before the intervention. Only medical staff who passed the training assessment could administer the clinical intervention. During the intervention process, research team members and the head nurse of the general surgery department jointly supervised the execution to ensure its effectiveness. They summarized implementation problems weekly and conducted continuous quality enhancements based on the identified causes.

**Table 2:** Intervention protocol for the intervention group and the control group.

Stage	Intervention group		Control group
	Objective	Measures	Measures
Preoperative	Optimize nutritional reserves	- Oral high-protein enteral nutrition formula for 5–7days preoperatively (400~600kcal/day,protein 1.5/kg/day) - Supplement with immunonutrients (ω-3 fatty acids 2g/day, glutamine 0.3 g/kg/day)	- No specific preoperative nutritional reinforcement.​
Intraoperative Management	Establish EN access	- Intraoperative placement of a nasoenteric tube	- Intraoperative placement of a nasoenteric tube
Early Postoperative (0–24h)	Initiate EN to reduce catabolism	- Initiate isosmotic EN within 24h postoperatively, starting at 20ml/h and increasing by 10ml/h every 12h	-Connect the nasoenteric tube to the negative pressure drainage device for gastrointestinal decompression. -Fasting
Late Postoperative (72h–7d)	Maintain nutrition and transition to oral diet	- Supplement with immunonutrients (ω-3 fatty acids 2g/day, glutamine 0.3 g/kg/day) - Gradual transition to oral diet starting on postoperative day 5 (with concurrent reduction in EN)	-Upon return of gastrointestinal function (passage of flatus) and removal of the gastrointestinal decompression tube, patients were instructed to sip water and consume 1–2 tablespoons of rice broth every 1–2h. -On Day 2 , a half-volume (50–80ml) liquid diet was administered every 2h. On Day 3, a full-quantity (100–150ml) liquid diet was introduced 5–6times daily. By Day 4, patients transitioned to a semi-solid diet.

### Data collection

#### Primary observational indicators

The rate of skeletal muscle mass loss on POD7 was calculated as follows:

Rate of skeletal muscle mass loss on POD7 = (Admission-POD 7)/Admission*100%.

The skeletal muscle mass measurement method involved the use of a body composition analyzer (Inbody 720, Inbody Co., South Korea), which operates on the principle of bioelectrical impedance. The device calculates muscle content in the body by determining impedance at different frequencies. Measurements were performed by ensuring that the patient was fasting or had not eaten for at least 2 h; instructing the patient to empty their bowels and bladder; and weighing the patient. Measurements were taken within 24 h of admission and on POD 7.

#### Secondary observational indicators

The rate of grip strength loss on POD 7 was determined as follows:

Rate of grip strength loss on POD 7 = (Admission-POD 7)/Admission*100%.

Grip strength, a primary indicator of hand force (particularly, upper limb strength) and human function, was assessed using an electronic grip dynamometer (EH101, CAMRY, Guangdong). This grip strength test adheres to the internationally recognized gold standard, as recommended by the American Society of Hand Therapists.

In this study, the patients sat with legs shoulder-width apart, hips and knees bent at 90°, upper arms close to the chest in a neutral position, elbows bent at 90°, forearms parallel to the ground, and wrists extended at 0°–30°, maintaining 0°–15° of ulnar deviation. Each hand was measured three times, with intervals of more than 15 s between measurements, and the average value was determined. Measurements were conducted within 24 h of admission and on POD 7.

(2)The rate of 6-meter walking speed loss on POD 7 was determined using the 6-meter walk test. Patients were required to walk a straight line of 6 m at their usual pace. The average time of two trials was recorded to calculate the walking speed over a distance of 6 m. Measurements were taken within 24 h of admission and on POD 7.

(3)The rate of weight loss on POD 7 was determined. Data were collected within 24 h of admission and on POD 7.

### Statistical analysis

All data were first checked for omissions and logical errors and then entered by two researchers using dual-core data entry. Statistical analysis was performed using IBM SPSS Statistics 22.0, with significance set at *p*<0.05. Quantitative data following normal distribution were expressed as mean ± standard deviation, and group comparisons were conducted using *t*-tests. For data not following a normal distribution, the median and interquartile range *M* (P25, P75) were used, and group comparisons were conducted using rank-sum tests. Categorical data were represented by frequency and proportion. When E≥5 and n>40, the Pearson chi-square test was used; when 1<E<5 and n>40, the continuity correction chi-square test was employed. When E<1 or n<40, Fisher’s exact test was used. Ordinal data were represented by frequency, and the nonparametric rank-sum test (Mann–Whitney *U* test) was used. Multiple linear regression analysis was used to control for confounding factors. Quantitative data not conforming to a normal distribution were analyzed by linear regression after logarithmic transformation. The corrected confounding factors included gender, age, education level, Nutritional Risk Screening 2002 (NRS2002) score, tumor stage, presence of comorbid diabetes, surgical method, operation time, and intraoperative blood loss.

## Result

### General details

Details of demographics, anthropometrics, and clinical characteristics are listed in **Table 1**. No significant differences in demographics, anthropometrics, and clinical characteristics were observed between the intervention and control groups. For the intervention group, the median age was 69 y (58.0, 75.0), and the proportion of female patients was 23.7%; for the control group, the median age was 70.5 y (60.8, 73.0), and the female sex distribution was 34.2%.

### Nutritional intervention outcomes​

Quantitative details of nutritional delivery are presented in **Table 3**. For the intervention group, preoperative EN supplementation averaged 582.9 ± 71.0kcal/day. Postoperatively, isosmotic EN (0.9% sodium chloride, 250ml) was initiated at 14.9 ± 4.4hours, and transitioned to formula feed within 38.9 ± 4.4hours. The control group relied exclusively on oral liquid intake following return of bowel function, with no EN support administered. Oral feeding was initiated at 69.5± 28.4hours postoperatively, achieving 107.4 ± 15.0ml within 24h post-flatus.

**Table 3:** Actual amount of nutritional intervention for the control and intervention groups.

Variable	Intervention group (n=38) (Mean ± SD)	Control group(n=38) (Mean ± SD)
Preoperative total daily EN supplement (kcal)​​	582.9 ± 71.0	–
Time to initiate isotonic EN (NS) postop (h)​​	14.9 ± 4.4	–
Time to transition from NS to formula EN (h)​​	38.9 ± 4.4	–
Total EN intake 48-72h postop (kcal)​​	630.0±32.6	–
Total EN intake 72-96h postop (kcal)​​	817.7±196.2	–
Time to initiate oral feeding after surgery(h)	–	69.5±28.4
Liquid intake within 24h post-flatus (ml)​​	–	107.4 ± 15.0
Liquid intake 24-48h post-flatus (ml)​​	–	503.2 ± 50.6
Liquid intake 48-72h post-flatus (ml)​​	–	931.3±108.7

### Change of observational indicators

**Figure 2:**
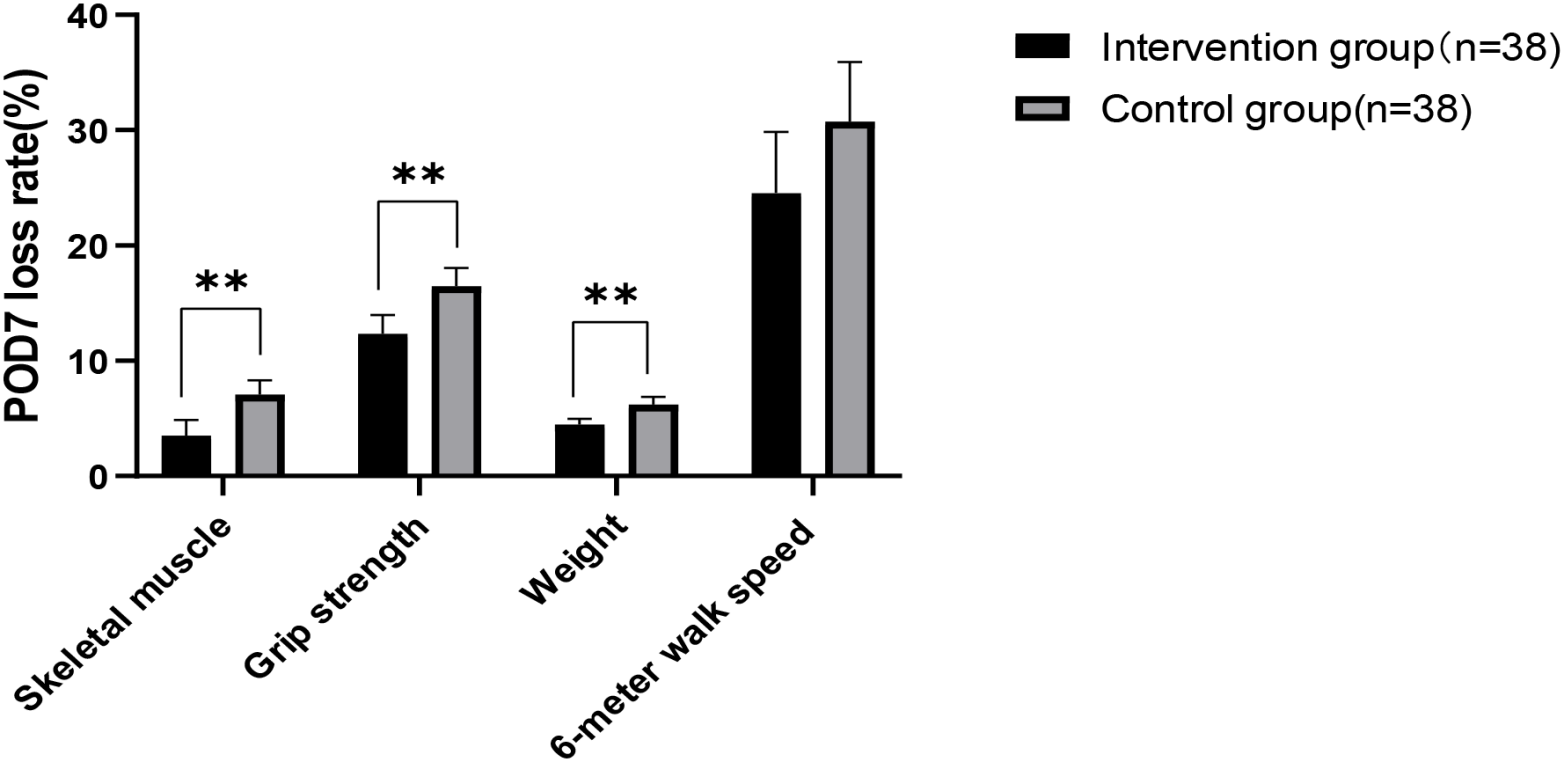
Analysis of changes in observational indicators. Significant differences between the intervention group and control group derived using the *t*-test (**: *p* < 0.01).

After the intervention, no significant differences were found in all indicators between the groups at baseline and on POD 7 (**Table 4**). All measured indicators exhibited a downward trend on POD 7. However, the rates of skeletal muscle mass, grip strength, and weight loss on POD 7 were lower in the intervention group than in the control group, with significant differences (*p*<0.01) (**Table 4**) (**Figure 2**). The effect of the evidence-based perioperative EN program intervention on the changes in observational indicators was evaluated based on the results derived from the multiple linear regression model, adjusted for age, gender, NRS2002, education level, diabetes comorbidity, tumor staging, surgical approach, intraoperative blood loss, and operation time, were used to evaluate (**Table 5**).

**Table 4:** Changes in observational indicators by Postoperative Day 7.

Outcome		Intervention group (n=38)	Control group (n=38)	*t/z*	*p*
Skeletal muscle mass	Baseline (kg, $\bar{x}\pm s$ )	24.8±3.9	25.2±4.2	-0.393	0.695^a^
	POD 7 (kg, $\bar{x}\pm s$ )	23.94±3.7	23.4±4.0	0.605	0.547^a^
	Loss rate on POD 7 (%, $\bar{x}\pm s$ )	3.5±4.1	7.1±3.8	-3.94	<0.01^a^
Grip strength	Baseline (kg), median (IQR)	24.6 (21.3,28.3)	24.7 (21.9,30.8)	-0.857	0.391 ^b^
	POD 7 (kg), median (IQR)	21.1 (17.9,26.0)	21.0 (18.1,25.0)	-0.057	0.954 ^b^
	Loss rate on POD 7 (%, $\bar{x}\pm s$ )	12.3±5.00	16.5±4.8	-3.671	<0.01^a^
6-meter walk speed	Baseline (m/s), median (IQR)	1.0 (0.9,1.1)	1.0 (0.9,1.2)	-0.843	0.399^b^
	POD 7 (m/s, $\bar{x}\pm s$ )	0.7±0.2	0.7±0.2	1.231	0.222 ^a^
	Loss rate on POD 7 (%, $\bar{x}\pm s$ )	24.6±16.1	30.8±15.7	-1.697	0.094 ^a^
Weight	Baseline (kg, $\bar{x}\pm s$ )	62.8±10.1	60.6±10.4	0.927	0.357 ^a^
	POD 7 (kg, $\bar{x}\pm s$ )	60.0±9.8	56.8±9.8	1.417	0.161 ^a^
	Loss rate on POD 7 (%, $\bar{x}\pm s$ )	4.5±1.6	6.2±2.0	-4.248	<0.01 ^a^

a represents the *t*-test, b represents the Mann–Whitney *U* test.

**Table 5:** Effect of the program for gastric cancer intervention on observational indicators, as determined using the multiple linear regression model.

Unadjusted						Adjusted		
Outcome	β (SE)	95%CI	t	*P*	β (SE)	95%CI	t	*P*
skeletal muscle mass loss rate on POD 7	0.416(0.907)	1.796–5.353	3.940	<0.01	0.464(0.464)	2.184–5.786	4.336	< 0.01
Grip strength loss rate on POD 7	0.392(1.126)	1.926–6.341	3.671	<0.01	0.401(1.113)	2.040–6.403	3.792	< 0.01
Weight loss rate on POD 7	0.443(0.411)	0.940–2.552	4.248	<0.01	0.14(3.322)	0.942–2.680	4.085	< 0.01
6-meter walk speed loss rate on POD 7	0.193(3.657)	-0.963–13.371	1.697	0.094	0.145(3.824)	-2.841~12.150	1.217	0.228

In the adjusted model, age, gender, NRS2002, education level, diabetes comorbidity, tumor staging, surgical approach, intraoperative blood loss, and operation time are controlled.

## Discussion

### Preoperative combined with early postoperative EN program improves postoperative acute muscle wasting and nutritional status in GC patients

Skeletal muscle mass and grip strength are closely related as primary indicators for assessing postoperative acute muscle wasting. Grip strength is an important measure for evaluating upper limb muscle strength, and the skeletal muscle mass directly affects its magnitude (16). Otsuji H et al. defined surgery-related muscle loss as a percent change in the total psoas muscle area of less than 5.0% (17). Huang DD et al. reported that 31.82% of GC patients experience a muscle mass loss exceeding 10% by POD 7 (3). This finding underscores the importance of monitoring and intervening to address muscle conditions after GC surgery. Muscle loss not only affects physical recovery but may also increase the risk of complications, prolong hospital stays, and higher costs (18).A systematic review demonstrates that severe postoperative skeletal muscle loss occurred in 25.7% of GC patients and was significantly correlated with poorer Overall survival (14). Skeletal muscle mass, grip strength, and body weight are important indicators for assessing the nutritional status of GC patients in the perioperative period. A systematic review study found no significant differences (*p*>0.05) in skeletal muscle mass between the group receiving oral nutritional supplements and the control group (19). However, the effect of EN on the rate of skeletal muscle mass loss within 7 d postoperatively was not examined. This study showed that in GC patients who received preoperative combined with early postoperative EN intervention, the mean loss rates of skeletal muscle mass, grip strength, and weight on POD 7 were 3.5%, 12.3%, and 4.5% in the intervention group and 7.1%, 16.5%, 6.2% in the control group. There was a significant difference between the two groups (*p*<0.01).

This finding indicates that the program effectively reduce acute postoperative skeletal muscle mass and weight loss, maintaining muscle function. Several potential mechanisms may explain this result:

First, the fragmented nutritional management provided to the control group lacked multidisciplinary coordination and a systematic approach. In contrast, the experimental group’s scientifically rigorous program featured dynamic, phase-specific adaptations across the perioperative continuum, enhancing its clinical utility and ensuring adequate nutritional support for GC patients. Preoperative EN optimized patients’ physiological reserves before surgery, establishing a foundation for accelerated postoperative recovery. Postoperative EN effectively maintained nutritional status following resection. Critically, during postoperative days 2–3 (48–72 hours), combined nutritional support delivered 630 ± 32.6kcal/day via EN and 850kcal/day via PN, achieving a total energy supply of 1,480kcal/day. This closely approximated the early-phase requirement baseline for gastrectomy patients (15–25kcal/kg/day) consistent with expert consensus endorsing hypocaloric provision in the immediate postoperative period (20).

Second, GC patients often experience weight loss and malnutrition due to the tumor or the treatment process before surgery. Postoperative impairment of digestive function and limited food intake lead to insufficient nutritional intake. Initiating EN within 24 hours after surgery can maintain intestinal mucosal integrity. EN is administered directly into the digestive tract to provide nutrients, helping maintain the structure and function of the intestinal mucosa, promoting the proliferation and repair of intestinal cells, thereby preserving the integrity of the intestinal barrier (21). A healthy intestinal barrier can effectively prevent bacterial and endotoxin translocation reducing systemic inflammatory responses (22) and protecting skeletal muscle mass from inflammatory damage.

Third, standardized EN management ensures that patients meet their nutritional requirements, ensuring that patients receive adequate nutrition. The EN formula contains essential amino acids, particularly branched-chain amino acids, which are crucial for skeletal muscle mass protein synthesis. Moreover, the energy provided by EN helps meet the increased metabolic demands postoperatively, reducing reliance on skeletal muscle mass for energy and decreasing muscle protein breakdown.The experimental group received a protein target of 1.5 g/kg/day during the intermediate postoperative phase. This aligns with evidence-based recommendations for GC patients, whose protein requirements typically range between 1.2–1.5 g/kg/day to 2.0 g/kg/day depending on clinical status and recovery goals (23). Adequate protein intake can activate the mTOR pathway, promoting muscle protein synthesis (24).

Fourth, The immunonutrients delivered via EN in this protocol specifically ω-3 fatty acids (2 g/day) and glutamine (0.3 g/kg/day) effectively protect skeletal muscle mass against catabolic stress. As highlighted by Triantafillidis, glutamine plays a vital role in maintaining nitrogen balance and enhancing protein synthesis (25). Furthermore, glutamine modulates immune function by reducing tissue glutathione levels while elevating plasma glutathione concentrations, promoting T lymphocyte proliferation, augmenting natural killer (NK) cell activity, and suppressing TNF-α secretion (25). Critically, TNF-α serves as a core inflammatory mediator that directly induces muscle atrophy through activation of downstream signaling pathways, including NF-κB, p38MAPK, and JAK/STAT (26).Otherwise ω-3 fatty acids reduce the synthesis of eicosanoids and the expression of inflammatory cytokines such as interleukin-6 (IL-6) (25). In inflammatory environments, pro-inflammatory cytokines like IL-6 directly promote muscle atrophy through pathways including NF-κB and p38MAPK (26). The French Society of Digestive Surgery (Société Française de Chirurgie Digestive, SFCD) recommends preoperative immunonutrition support even for well-nourished GC patients. Such interventions in nutritionally adequate abdominal surgery patients improve outcomes by reducing postoperative infections, shortening hospitalization, and decreasing medical costs (27). Kabata et al. further reinforced this in a prospective randomized controlled trial, demonstrating that preoperative nutritional support in non-malnourished oncology patients maintains nutritional status and reduces the incidence and severity of postoperative complications (28). Collectively, these findings suggest that even patients with adequate baseline nutrition may experience underlying disease-induced metabolic alterations; when compounded by surgical stress, these changes can precipitate malnutrition and poorer outcomes. Thus, preoperative nutritional intervention may optimize metabolic resilience, though validation through multicenter, large-sample randomized controlled trials (RCTs) remains imperative. Concurrently, a systematic review reveals substantial heterogeneity in current immunoenutrition research: while only 45.5% of the included studies initiating interventions postoperatively reported definitive clinical benefits, 80% of the included studies indicated that the perioperative application of immunoenutrition provided some benefit for patients with GC undergoing gastrectomy (29), underscoring the need to standardize immunoenutrition formulations and intervention durations while developing tailored protocols for distinct nutritional risk populations.

Fifth, postoperative patients often rest in bed, potentially leading to rapid muscle loss due to lack of exercise. Meanwhile, malnutrition can accelerate muscle breakdown, affecting body weight and handgrip strength. Timely and effective individualization setting of energy target requirements, particularly for protein, can maintain nitrogen balance, slow down muscle loss, and facilitate postoperative muscle function recovery and rehabilitation in patients. In summary, implementing a perioperative EN program is crucial for improving postoperative acute muscle wasting and nutritional status in GC patients. Adequate EN support can enhance patient recovery and survival, reduce complications. Therefore, perioperative EN management programs should be actively promoted and applied in clinical practice to promote patient recovery and health.

### The program failed to improve the walking activity ability of GC patients after surgery

The 6-meter walking speed is an important indicator for assessing physical body function, which is closely related to skeletal muscle mass strength and mass. In diagnosing sarcopenia, the 6-meter walking speed is often used as one of the assessment criteria (30). The results of the current study indicate that the mean loss rate of the 6-meter-walk speed on POD 7 was 24.6% in the intervention group and 30.8% in the control group. No significant difference was found between the two groups (*p*>0.05). Postoperative 6-meter walking speed is influenced by various factors:

First, the physical condition of patients, including muscle strength and cardiopulmonary function, plays a crucial role in their postoperative walking ability. Second, the effect of the surgery cannot be overlooked. The type and extent of the procedure, along with patient recovery, can influence mobility. Third, pain management is a critical factor that can limit postoperative activity (31). If pain is not well-controlled, it can restrict movement, consequently affecting walking speed. The EN program may need to be integrated with effective pain management strategies to optimize outcomes. Psychological factors, such as anxiety and depression, can also hinder patient recovery and ability to stay active. Last, complications such as infections or thromboembolic events can also affect patient recovery and mobility. If not managed promptly and effectively, these issues can overshadow the benefits of EN. In conclusion, while EN is a vital component of perioperative care, walking speed is determined by multiple factors. A comprehensive approach that includes personalized EN program, pain management, psychological support, social engagement, and proactive complication management is necessary to improve walking speed in GC patients after surgery. Future studies should explore these factors in greater detail to develop more effective perioperative intervention programs.

### Limitations

This study has several limitations. First, the small sample size and single-center design limit its representativeness. In future studies, the program should be implemented in a multicenter setting. Second, the enrollment times of the control and experimental groups in this study are inconsistent, which may introduce a selection bias and affect the validity of the research results. The research results should be interpreted with caution. Future randomized controlled studies can be conducted to further clarify the effectiveness of the program. Third, the program does not distinguish the effects of different surgical methods or surgical approaches on the use of EN in GC patients. The need for EN may depend on the surgical method (total or partial gastrectomy, and the surgical approach (open surgery, laparoscopic surgery, or robotic surgery. Future research should clarify the effectiveness of different EN management methods for various surgical methods and approaches. The final limitation of this study is the relatively short observation period. This brief time frame may prevent the observation of long-term effects or outcomes, potentially affecting the accuracy and generalizability of the findings. Future studies with extended observation periods are necessary to elucidate the long-term implications and effectiveness of the interventions or phenomena under investigation.

## Conclusions

This study develops a perioperative EN program for GC that helps enable medical staff to quickly and comprehensively obtain relevant information and provide a more comprehensive and holistic EN program for GC patients. This program helps reduce postoperative acute muscle wasting, grip strength loss, and weight loss, and also demonstrates clinical value for promoting its broader implementation

## Data Availability

The datasets presented in this study can be found in online repositories. The names of the repository/repositories and accession number(s) can be found below: http://www.medresman.org.cn/pub/cn/proj/projectshshow.aspx?proj=6148.
